# Age-related iron accumulation and demyelination in the basal ganglia are closely related to verbal memory and executive functioning

**DOI:** 10.1038/s41598-021-88840-1

**Published:** 2021-05-03

**Authors:** Davina Biel, Tineke K. Steiger, Nico Bunzeck

**Affiliations:** 1 Department of Psychology, University of Lübeck, 23562 Lübeck, Germany; 2 Institute for Stroke and Dementia Research (ISD), University Hospital, LMU Munich, 81377 Munich, Germany; 3 Center of Brain, Behavior and Metabolism (CBBM), University of Lübeck, Ratzeburger Allee 160, 23562 Lübeck, Germany

**Keywords:** Cognitive ageing, Cognitive neuroscience, Neural ageing, Biomarkers

## Abstract

Age-related cognitive decline has been linked to alterations of the dopaminergic system and its subcortical trajectories. Recent work suggests a critical role of iron accumulation within the basal ganglia (BG) in verbal memory performance, and increased iron levels have been related to demyelination. However, the specificity of age-related iron increases with respect to cognitive functions remains unclear. Therefore, we investigated the interplay of age, cognitive performance, and structural integrity of the BG. In total, 79 healthy older participants underwent a broad cognitive assessment (fluid and crystallized intelligence, verbal and numeric memory, processing speed, executive functions) and structural MRI. As expected, performance in most cognitive tests had a negative relationship with age. Moreover, BG grey matter volume and magnetization transfer (MT, indicative of myelin) decreased, and R2* (indicative of iron) increased with age. Importantly, R2* and demyelination negatively correlated with verbal memory and executive functions. Within the SN/VTA, age correlated negatively with MT, but there was no clear evidence in favor of a relationship between behavior and R2* or MT. Our results suggest that age-related increases in iron and demyelination within the BG, which are part of a fronto-striatal network, not only impact on verbal memory but also executive functions.

## Introduction

Cognitive declines in healthy aging have previously been linked to cortical and subcortical degeneration (e.g.,^1^), but the underlying microstructural changes are poorly understood. In this regard, recent studies could show that iron accumulations within the basal ganglia (BG) closely relate to demyelination and deficits in verbal long-term memory performance^2,3^. The specificity of BG iron accumulations on cognitive functioning, however, remains unclear.

In contrast to grey matter (GM) and myelin, iron accumulations can mainly be observed in subcortical structures^4–6^. Within the BG and interconnected substantia nigra/ventral tegmental area (SN/VTA), increased iron levels are typically related to motor problems in neuropsychiatric diseases (i.e., Parkinson’s disease;^7,8^), but they are also a hallmark of healthy aging^9^. Importantly, iron accumulations have been further related to demyelination^10,11^, and a recent study observed a negative association between iron levels and myelination within the striatum^3^.

At the cellular level, glia cells (i.e., oligodendrocytes) require iron for myelin production^12^, but as iron accumulates, oxidative stress can damage the myelin sheaths^13^. Besides its role in myelin production, the non-heme iron enzyme tyrosine hydroxylase is an important factor for the synthesis of dopamine (DA)^14^. However, elevated iron levels within the SN/VTA block DA production and even promote cell death^8,14^. Indeed, in Parkinson’s disease, which is characterized by increased iron levels^15^, DA transport is reduced within the SN/VTA^16,17^ and further accompanied by a volume loss^18^. In healthy older adults, SN/VTA iron levels do not appear to be increased^19,20^, but the structural integrity of the SN/VTA, as measured with magnetization transfer ratio (MTR, a marker of myelination;^21^) was reduced and predictive of verbal memory performance^22^. However, the relationship of iron and myelin within the SN/VTA in healthy aging remains little understood.

Within the brain, iron can be observed in two different forms: heme iron and non-heme iron. While heme iron is functionally linked to the hemoglobin molecule, and therefore exclusive within circulating or accumulating blood; non-heme iron is present within virtually all brain cells^4,23^ and involved in numerous metabolic functions^24^. Importantly, MRI based structural measures of iron, such as R2* that we employed here, are supposed to reflect non-heme iron. For a more detailed review see^24^.

Apart from age-related iron accumulations, decreases in GM volume^25^ and myelin^26,27^ have been reported. GM volume loss is typical throughout the cortex, including frontal brain regions^27,28^, but it can also be observed within the hippocampus^29^, putamen^27^, and caudate ^9,30^. Age-related demyelination, on the other hand, has been found within white matter (WM) structures, including frontal and parietal regions, the optic radiations, the corpus callosum, and the corticospinal tract ^27,28^. A decrease of myelin within GM has been described within the thalamus, Heschl’s gyri, the caudate nucleus, and the cerebellum^27^.

Previously, the integration of age, structural changes, and cognitive decline has been discussed (see as a review^31^). The scaffolding theory of aging and cognition (STAC)^32^ provides a framework for brain-behavior associations, suggesting that biological aging affects brain structure, which in turn impacts on cognitive performance. Although an age-related decrease of cognitive performance has been widely established^1^, several questions regarding the underlying mechanisms remain open. For instance, typical increases of inter-individual variability in cognition within the older population indicate that age alone may not explain cognitive decline^32^, and it suggests that individual differences in structural integrity need to be considered (see also^35–37^). While we expect that age has a direct effect on brain structure and hence, cognitive performance, we hypothesize that individual differences in structural integrity may impact on cognition, which can be independent of age.

Here, we conducted a detailed cognitive assessment (i.e., fluid and crystallized intelligence, verbal memory, numeric memory, processing speed, and executive functions) to further investigate the links between age, cognitive performance and in vivo structural integrity of the BG in healthy older adults. We performed voxel-based morphometry (VBM) to quantify GM volume, and voxel-based quantification (VBQ) to examine the magnetization transfer saturation (MT) and the effective transverse relaxation rate (R2*). Both markers, MT and R2*, strongly correlate with myelin and iron concentrations, respectively, as revealed by post mortem studies^21,38^. First, we expected a negative association between age and cognitive performance. Regarding age and brain structure, we had three major hypotheses: (a) GM volume decreases^25^, (b) MT decreases^26^, and (c) R2* increases^27^. In addition, we expected a negative correlation between R2* and MT within the BG^3,26^. In terms of brain-behavior associations, we hypothesized correlations between structural integrity (a-c) and cognitive performance even when controlling for age. Specifically, we expected a positive relationship between cognitive performance and GM volume as well as cognitive performance and myelin. In contrast, for iron accumulations, a negative link to cognitive performance was hypothesized. Finally, a voxel-based region of interest (ROI) analysis was performed for the SN/VTA to investigate the association between MT, R2* levels and cognitive performance. Here, we tested whether the hypotheses for the BG also apply to the SN/VTA.

## Materials and methods

### Experimental design, procedure, and participants

All participants were part of a cognitive training study, which included a test at baseline (t1), a four-week cognitive training, and a follow up examination (t2) at the University of Lübeck. At both time points, all participants received a detailed neuropsychological assessment (see below) and a structural MRI scan (see below). For the current study, only baseline data (t1) were analyzed. The findings of the cognitive training study are reported elsewhere^39^. Note that the methods sections of cognitive assessment, image acquisition, and VBM/VBQ processing are adapted from^39^.

In total, 92 healthy, right-handed, German speaking older adults were recruited. However, nine participants were excluded due to a history of neurological, psychological or other severe physical disorders, drug abuse, CNS affecting medication intake (less than 2 weeks before testing), non-removable metal implants or claustrophobia. Four additional participants had to be excluded due to technical issues or structural abnormalities observed in the MRI data. Moreover, participants were excluded with > 5 points in the Geriatric Depression Scale (GDS, max. 15 points, > 5 points indicates mild depression;^40^) and < 22 points in the Montreal Cognitive Assessment (MoCA, max. 30 points;^41,42^). A value of 22 was chosen based on a study by^42^, suggesting that it might be an appropriate cut-off for mild cognitive impairment (MCI). Finally, 79 participants (age range 50–80 years, mean age 63.54 ± 8.48, 39 females) completed the MRI sessions and could, therefore, be included in further analyses. Within this group, the following numbers of participants per cohort were included: 50–60 years n = 32, 61–70 years n = 28, 71–80 years n = 19.

Participants were recruited through announcements in the local newspaper or the database of the University of Lübeck^43^. All participants signed a written informed consent and received monetary compensation. The study was approved by the local ethical committee of the University of Lübeck, Germany, and in accordance with the Declaration of Helsinki.

### Cognitive assessment

For cognitive assessment, the German Leistungsprüfsystem (LPS 50 + ^44^), the German Mehrfachwahl-Wortschatz-Test (MWT^45,46^), the verbal learning memory test (VLMT^47^), the digit span^48^, the d2-R^49^, and the trail making test (TMT^50^) were applied.

Intellectual functioning (i.e., fluid intelligence; Gf) was measured by the LPS 50 + short version (for people aged 50–90 years), which includes a battery of time restricted paper pencil tasks (duration ~ 30 min). For crystallized intelligence (Gc), the MWT was applied; it provides 37 rows, each containing four pseudo-words and one correct word, which has to be identified (with no time restriction).

Verbal memory was examined using the VLMT. Here, a word list of 15 nonrelated items was verbally presented for five subsequent times. Each time, participants were asked to recall as many words as possible. Recalled words were noted from the examiner (total sum of correctly recalled words of all five runs refers to VLMT learning in further analysis). In a sixth run, an interference list of 15 words was verbally presented, which had to be immediately recalled. Subsequently, participants were asked to recall words from the initial list (without further verbal presentation from the examiner). After 20 min (again without further verbal presentation), the initial word list had to be recalled (VLMT free recall). Consolidation loss (VLMT cons) is calculated by subtracting the amount of words remembered in the fifth round from VLMT free recall. Finally, a recognition task was conducted by presenting the initial word list intermixed with words from the interference list and previously not presented new words. The list was read out aloud and participants had to judge whether they recognized a word from the initial word list or not (VLMT recognition).

Numeric short-term memory was assessed by using a digit span forward and backward test (forward: starting with 3 digits, ending with 8 digits or after 2 errors within the same difficulty level; backward: starting with 2, ending with 7 digits or after 2 errors within the same difficulty level). Subsequently, scores of digit span forward and backward were summed up.

Processing speed and attention was tested using the standardized d2-R test. Here, participants had to mark as many targets (d’s with exactly two dashes placed above or under the d) as possible within 14 rows containing d and p letters. After 20 s, participants had to switch to the next row. Following to the test manual, the first and last row were not included into the analysis. After 4.6 min the task was completed. KL (i.e., concentration) is calculated by subtracting false positives and omissions from the total amount of marked items.

The TMT is a cognitive assessment consisting of two parts, TMT-A (sustained attention) and TMT-B (divided attention). In TMT-A, participants had to connect circles containing numbers as fast as possible into the right order (e.g., 1–2–3–4), while in TMT-B, circles containing numbers and letters had to be connected in alternating order (e.g., 1-A-2-B-3-C). Although both TMT subtests have a strong visual search component and are highly correlated^51^—probably due to the involvement of overlapping cognitive processes (e.g., processing speed, visual search)—the TMT-B is cognitively more demanding^52,53^. While the TMT-A has been predominantly linked to motor speed, the TMT-B demands higher cognitive processes such as cognitive flexibility^54–56^ and has been linked to executive control/function^57^. Following standardized test instructions, participants were made aware of mistakes. The resulting additional time is reflected in the total time needed for solving the task. For the regression analyses, the required time for both TMT-scores were summed up (TMT-sum) in order to reduce statistical complexity. In order to unfold the different components of the TMT, post hoc analyses were performed. Here, the separate TMT-A and TMT-B scores, as well as TMT-difference (TMT-diff) and TMT-ratio were included. TMT-diff (TMT-B minus TMT-A) and TMT-ratio (TMT-B divided TMT-A) have been previously used to provide an index for executive functioning by disentangling the motor speed component of TMT-A from the more complex TMT-B^53,57^. Besides the TMT-diff score, which has also been linked to cognitive efficiency^58^, the TMT-ratio score controls for intra-individual factors^57,59,60^, which found implication in the assessment of cerebral dysfunction and impairments^59,61^.

### Image acquisition

Structural MRI was performed at the University of Lübeck using a 3 T Siemens Magnetom Skyra scanner equipped with a 64-channel head coil. Whole-brain multiparameter mapping (MPM) was acquired as reported in previous studies^62,63^ using multi-echo 3D fast low angle shot (FLASH) at 1 mm isotropic resolution. The volumes were acquired for T1, proton density (PD), and magnetization transfer (MT) weightings. The weightings differed in TE, TR, and flip angles. T1-weighted: six echo times (TE = 2.2, 4.7, 7.2, 9.7, 12.2, 15 ms), TR = 19 ms, flip angle = 20°; PD-weighted: eight echo times (TE = 2.2, 4.7, 7.2, 9.7, 12.2, 15, 17.5, 20 ms), TR = 24 ms, flip angle = 6°; MT-weighted: six echo times (TE: 2.2, 4.7, 7.2, 9.7, 12.2, 15 ms), TR = 37 ms, flip angle = 6°. A Gaussian MT-pulse following Siemens product sequences was applied. To shorten the scan duration, a partial Fourier 6/8 was used. Parallel imaging with a GRAPPA acceleration factor of 2 was applied. The total scanning time of the MPM protocol was approx. 20 min.

Subsequently, two runs of diffusion weighted imaging (DWI) using an EPI sequence were performed during the same scanning session (scanning time approx. 16 min). The images were later used for further analysis (results will be reported elsewhere).

MR data were further processed on the Statistical Parametric Mapping framework (SPM 12, Wellcome Trust Center for Neuroimaging, London) and MATLAB software (R2014b version, Mathworks). R2* maps were calculated through a regression of the log signal from the PD-weighted echoes. Averaging the set of echoes for each weighting increased the signal-to-noise-ratio for estimation of the MT map^64^. The semiquantitative MT map was calculated as described by^65,66^. Subsequently, to ensure uniform orientations, images were slightly manually re-orientated according to individual posture during the MRI acquisition using SPM Check Reg and Display options^67^.

### Voxel-based morphometry and voxel-based quantification

GM volumes were processed and analyzed following a protocol for voxel-based morphometry using SPM’s batch system (VBM;^67,68^). Since MT maps provide increased contrast for subcortical regions^69,70^, in a first step, they were used for segmentation of the different tissue groups. Subsequently, images of GM, WM, and cerebrospinal fluid (CSF) were generated in native space^71^. Applying high dimensional warping, images were then normalized to MNI space using the diffeomorphic registration algorithm (DARTEL) implemented in SPM, scaled by the Jacobian determinants of the deformation field and smoothed with an isotropic Gaussian Kernel of 6 mm full width at half maximum (FWHM). Finally, the resulting smoothed, modulated and normalized GM images were used for statistical analysis.

Voxel-based quantification (VBQ) analysis provides sensitivity to tissue microstructure and is therefore well suited to test differences in R2* and MT, which are sensible marker for alterations in subcortical brain regions. VBQ was processed using the open source hMRI toolbox^72^ embedded in the SPM framework. Using the integrated processing pipeline of the toolbox, the previously generated MT maps were further processed using the modules tissue segmentation (GM, WM, and CSF), DARTEL, creation of templates, and normalization to MNI space. Subsequently, tissue-weighted smoothing with a FWHM isotropic Gaussian kernel of 3 mm^28^ was performed. Resulting images of R2* and MT for GM were used to indirectly test for differences in iron levels (i.e., R2*) and myelination (i.e., MT) of brain tissue (see also^27,28^). Note that we did not analyze WM subspace, since we focused on the BG structures that typically do not contain much WM^73^. Moreover, the estimation of iron concentrations in WM regions (on the basis of quantitative susceptibility mapping) appears to be less accurate^74^. Analyses of MTR within GM structures have been related to demyelination^75^ and cognitive impairment^76^, suggesting a link between behavior and MT integrity of subcortical structures.

### Manual segmentation of the SN/VTA

**Figure 1:**
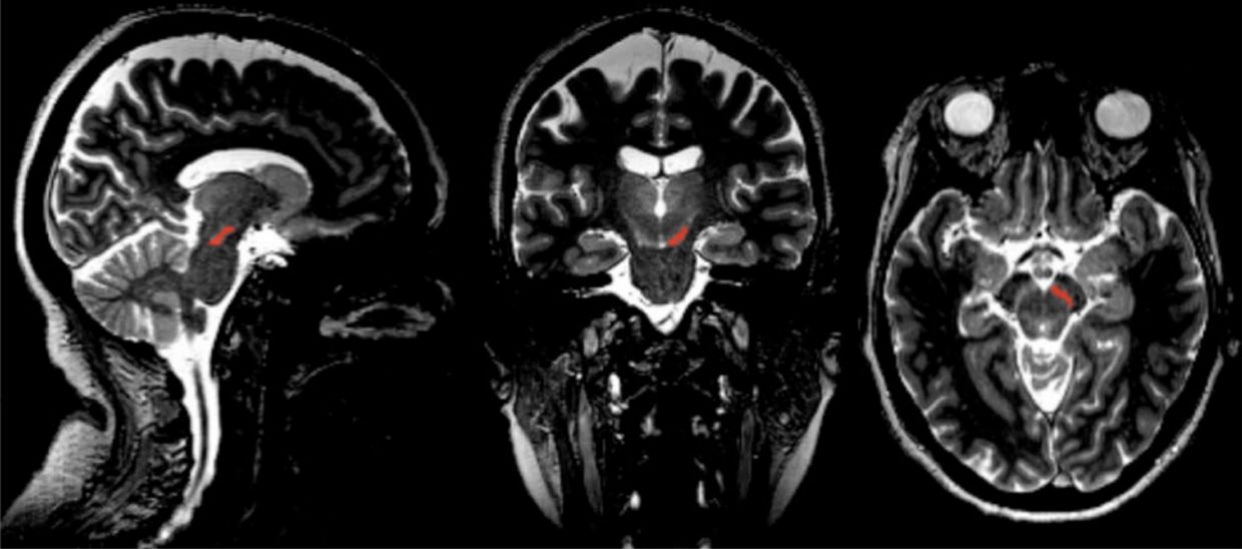
Manual segmentation of an individual SN/VTA. Within the midbrain, the SN/VTA can be identified as bright stripe on MT-weighted images. Bilateral SN/VTAs were defined as ROIs, which were used to quantify mean MT and R2* values. For illustration purposes, only the right SN/VTA is marked in red (i.e., ROI).

The SN/VTA is a fairly small subcortical structure and inter-individual differences of the SN/VTA boundaries can account for inaccuracy when applying normalized anatomical masks. Therefore, in the present study, a voxel-based region of interest (ROI) analysis in native space was conducted. Following previous studies^22,77^ and using MRIcron tools, individual SN/VTAs were segmented based on the intense contrast change between the bright grey color and the dark grey color of the adjacent tissue in the MT-weighted image (Fig. 1). The upper limit of the SN/VTA-ROI was selected at a level of the superior colliculi. The anterior part of the SN/VTA-ROI was limited by the interpeduncular fossa and posterior borders were limited by the lateral side of the cerebral peduncle. The lower limit of the SN/VTA-ROI was identified as the last even grey colored cross-sectional area. The total rostrocaudal extension of the ROI included approx. 10 slices depending on the individual size of the SN/VTA.

All segmentations were performed twice by one person. Only the latter segmentations were used for further analyses. Subsequently, the ROIs were extracted and projected as an overlay on the corresponding MT and R2* images to obtain the mean values (see also^22,78^). Reliability of the segmentation was tested with an intra-class correlation coefficient (ICC;^79^) using the ICC command within R statistical software^80^ (see http://personality-project.org/r/html/ICC.html). There were significant correlations between the first and the second manual segmentation regarding values for MT (ICC = 0.98, p < 0.001) and R2* (ICC = 0.99, p < 0.001), suggesting high intra-rater reliability. To test inter-rater reliability, a second person performed the SN/VTA segmentation. Subsequently, segmentations of both examiners were correlated showing high inter-rater reliability for MT (ICC = 0.98, p < 0.001) and R2* (ICC = 0.99, p < 0.001). Note, that the SN/VTA had to be manually segmented since the Harvard–Oxford-Atlas does not provide it as ROI.

### Statistical analyses

Participants with more than 3 SD above or below the overall mean of a specific neuropsychological test were excluded from the respective analysis. For analyses of the relationship between microstructure and cognitive functioning, multiple regression analyses for VBM and VBQ were calculated with SPM using Matlab software. Covariates were age, LPS 50 + , MWT, VLMT learning, VLMT cons, VLMT free recall, digit span, and d2-R. Due to one missing value in the VLMT recognition test and two outliers (> 3 SD) in the TMT, two additional linear regression analyses of VBM and VBQ were conducted for age and VLMT recognition, and age and TMT.

Effects within subcortical regions of interest (ROI) were investigated using a small volume correction (SVC) with a BG mask (i.e., caudate, pallidum, putamen, nucleus accumbens). The mask was taken from the Harvard–Oxford-Atlas (50% probability mask), implemented in the FMRIB Software Library (FSL;^81^). Clusters with at least 50 voxels and a p < 0.05 after familywise error correction (FWE) at the cluster level (p < 0.001 uncorrected at peak-voxel level) were regarded as significant. For post hoc analyses and depictions of effects, MT and R2* values were extracted from significant brain regions (see “Results”). To further examine effects of age on cognition, a post hoc partial correlation for age was applied for significant results of the VBM and VBQ analyses. In addition to the SVC analysis, whole brain analysis (cerebellum excluded) was performed for all regression models on GM subspace (FWE, p < 0.05, k = 50 voxel), to test whether relationships between structure and cognition are specific to the BG.

To obtain a clearer picture about the age-structure-behavior relationship, a mediation analysis was performed for significant structure-behavior correlates with age as predictor, structure as mediator, and cognitive performance as dependent variable. The mediation analysis was performed with the TMT-diff score (instead of TMT-sum), since it provides a purer measure of executive functioning compared to the TMT-sum score. Moreover, in post-hoc calculations of the MRI regression analysis only TMT-diff but not TMT-ratio became significant; therefore, no further analyses with TMT-ratio were performed here. The analysis was performed using the medmod module (see https://cran.r-project.org/web/packages/medmod/medmod.pdf) within R statistical software yielding indirect, direct, and total effects. Z-transformation was performed.

Finally, SN/VTA values (MT and R2*) and their relationship to cognitive performance were investigated using a correlation analysis in SPSS. MT and R2* values were averaged across voxels for each individual SN/VTA-ROI (see above). The correlation analysis of the structural parameter (MT, R2*), age and the neuropsychological tests was Benjamini-Hochberg (FDR) corrected. To test whether significant correlations with age or cognitive tests within the BG are different from those within the SN/VTA, a comparison of correlation coefficients for dependent groups with one overlapping variable (i.e. age, cognitive tests) was performed^82^. Analysis was computed using the cocor command (cocor.dep.groups.overlap) within R statistical software (see https://CRAN.R-project.org/package=cocor;^83^).

Not all data were normally distributed; however, due to the large sample size (79 participants), our inferential statistics are supposed to be robust against a violation of the assumption of normality (i.e., central limit theorem;^84^).

## Results

### VBM analysis

A multiple regression analysis was computed, including the covariates age, LPS 50 + , MWT, VLMT learning, VLMT cons, VLMT free recall, digit span, and d2-R. Due to outliers/missing values in the VLMT recognition and TMT, two additional regression analyses were conducted for age and VLMT recognition, as well as age and TMT. Therefore, three separate regression analyses were performed; the effects were small volume corrected, using a BG mask (cluster level FWE, p < 0.05; see “Materials and methods”).

**Figure 2:**
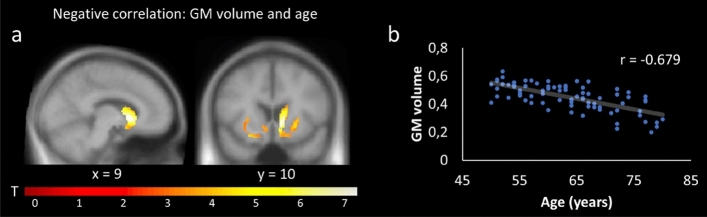
Negative relationship between age and GM volume. (**a**) SPM showing a significant negative relationship between GM volume and age within the right caudate, pallidum, and putamen; (**b**) showing the corresponding correlation plot for the cluster. For display purposes, SPMs were thresholded at p < 0.001, uncorrected, and superimposed on the mean T1-weighted image.

The VBM multiple regression analyses revealed a negative relationship between age and GM volume within the right caudate, pallidum, and putamen (Table 1; Fig. 2a-b), and a positive correlation between GM volume and VLMT recognition in the caudate (Table 1). There were no further significant correlations between GM and behavior. When adding age as a covariate (i.e., post hoc partial correlation), the effect between GM volume and VLMT recognition only reached trend level (r = 0.208, p = 0.07). No significant correlates between structure and cognition could be observed on subsequent whole brain analysis (FWE, p < 0.05, k = 50 voxel).

**Table 1:** Results of VBM and VBQ. Table shows results of VBM and VBQ (on R2* and MT GM) multiple regressions. Positive effects (one sided) are marked with a (+) and negative effects with a (−). P-values correspond to the cluster (FWE corrected) and the MNI coordinates to the respective peak voxel. Note that for VLMT recognition and the TMT separate regression analyses were performed (see text).

Analysis	Side	Region	p-value (FWE-corrected)	Number of voxels	MNI coordinates (mm)		
				k	x	y	z
**GM volume**							
Age (−)	R	Caudate	< 0.001	2132	9	10	2
VLMT recognition ( +)	R	Caudate	0.047	709	18	−21	22
GM R2*							
Age (+)	L	Putamen	< 0.001	766	−28	−6	0
	R	Putamen	< 0.001	544	28	0	2
VLMT recognition (−)	L	Putamen	< 0.001	1213	−30	−20	−2
	R	Putamen	< 0.001	998	30	−17	−3
VLMT free recall (−)	L	Putamen/Pallidum	0.001	168	−20	−6	6
	R	Putamen/Pallidum	< 0.001	450	23	−11	5
TMT-sum (+)	L	Putamen	< 0.001	194	−30	−20	−1
	L	Pallidum	0.003	153	−19	−10	1
	R	Putamen	0.001	189	32	−17	−3
TMT-B (+)	R	Putamen	< 0.001	233	31	−18	−4
	L	Pallidum	< 0.001	229	−16	−8	−4
	L	Putamen	0.001	187	−30	−20	−1
TMT-diff (+)	L	Pallidum	0.01	131	−16	−7	−5
**GM MT**							
Age (−)	L	Caudate	< 0.001	10,552	−17	13	6
	R	Caudate	< 0.001	2793	9	5	11
	R	Pallidum	< 0.001	6219	23	−3	2
VLMT recognition (+)	L	Caudate	< 0.001	1633	−13	5	21
	R	Caudate	< 0.001	1603	13	20	11
	L	Pallidum	0.001	133	−19	0	−5
VLMT free recall ( +)	L	Pallidum	0.04	73	−16	−4	−3
TMT-sum (−)	L	Pallidum	< 0.001	1901	−17	2	−3
	L	Caudate	< 0.001	1670	−13	5	21
	L	Putamen	0.001	136	−21	11	−2
	L	Putamen	0.013	92	−29	−11	0
	R	Pallidum	< 0.001	721	20	2	−2
	R	Caudate	0.005	106	14	−9	19
	R	Caudate	< 0.001	157	10	8	16
TMT-A (−)	L	Pallidum	< 0.001	643	−16	4	−2
	R	Pallidum	< 0.001	248	20	2	−1
	R	Putamen	0.028	83	25	8	−3
TMT-B (−)	L	Pallidum	< 0.001	1699	−17	2	−3
	L	Caudate	< 0.001	1067	−13	5	21
	R	Pallidum	< 0.001	363	21	2	−2
	L	Caudate	< 0.001	143	−13	−11	19
	R	Caudate	0.001	123	10	8	16
	R	Caudate	0.011	94	14	−9	19
	L	Putamen	0.019	87	−21	11	−2
TMT-diff (−)	L	Pallidum	< 0.001	538	−18	0	−6

### VBQ analysis on R2*

Similar to the VBM analysis, a multiple regression analysis on GM R2* was performed, including the covariates age, LPS 50 + , MWT, VLMT learning, VLMT cons, VLMT free recall, digit span, and d2-R. Due to outliers/missing values in the VLMT recognition and TMT, two additional regression analyses were conducted for age and VLMT recognition, as well as age and TMT. Therefore, three separate regression analyses were performed; the effects were small volume corrected, using a BG mask (cluster level FWE, p < 0.05; see “Materials and methods”).

**Figure 3:**
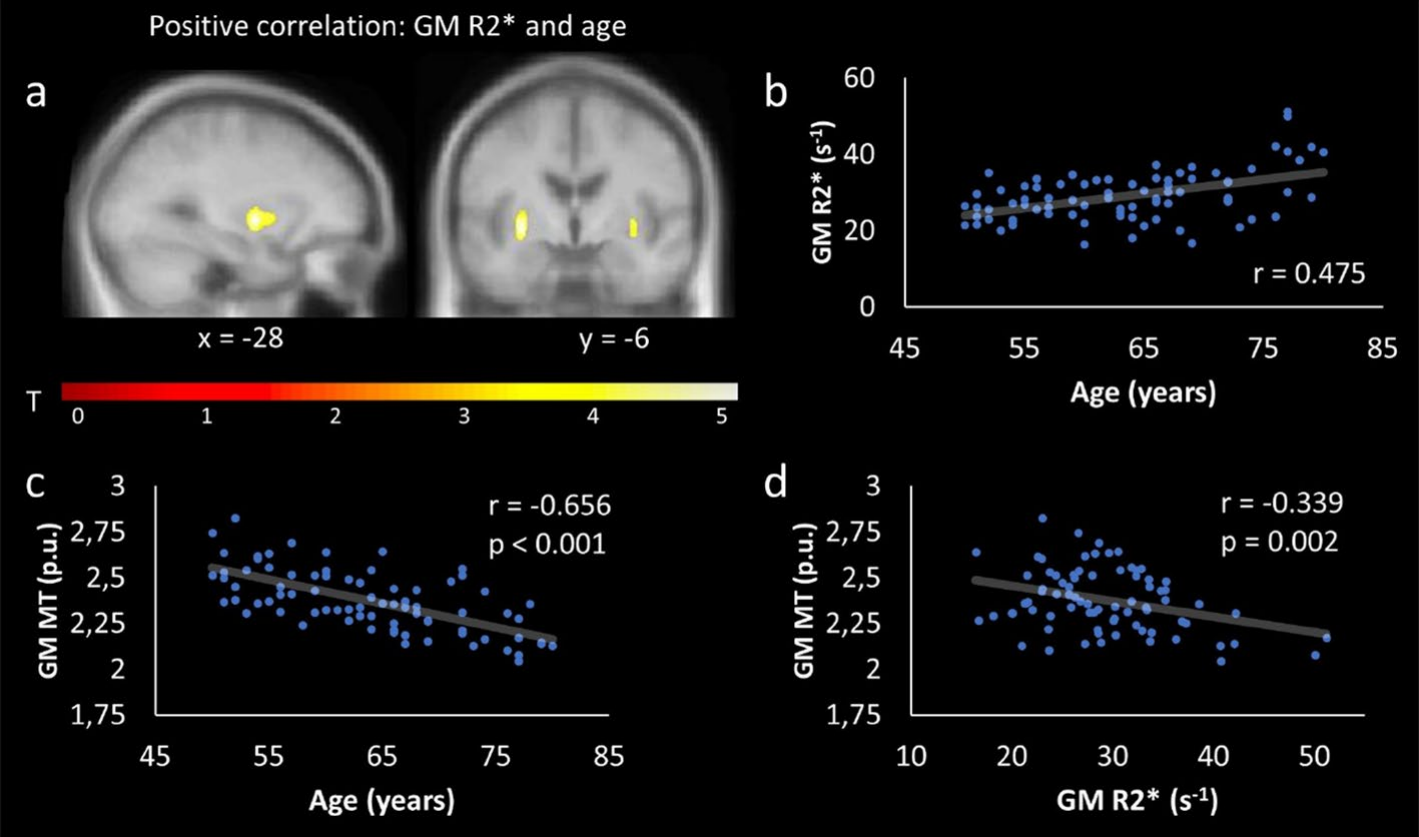
Positive relationship between age and GM R2*. (**a**) SPM showing a significant positive relationship between GM R2* and age within the left and right putamen; (b) shows the corresponding correlation plot for the cluster. For post hoc analyses, GM MT values were extracted from the same cluster. They revealed a negative relationship between GM MT and age (**c**), and a negative relationship (not corrected for age) between GM R2* and GM MT (**d**). For display purposes, SPM was thresholded at p < 0.001, uncorrected, and superimposed on the mean T1-weighted image.

The analyses showed a significant positive effect between age and R2* within the left and right putamen (SVC, cluster level FWE, p < 0.05; Table 1; Fig. 3a). Post hoc, MT and R2* values were extracted from that cluster for further analyses. As expected, there was a significant positive correlation between age and GM R2* (r = 0.475; Fig. 3b), a negative correlation between age and GM MT (r = −0.656, p < 0.001; Fig. 3c), and a negative correlation between GM MT and GM R2* (r = −0.339, p = 0.002; adjusted alpha level: 0.05/3 = 0.016; Fig. 3d). However, when adding age as covariate in a subsequent partial correlation, the relationship between GM MT and GM R2* no longer remained significant (r = −0.042, p = 0.717).

**Figure 4:**
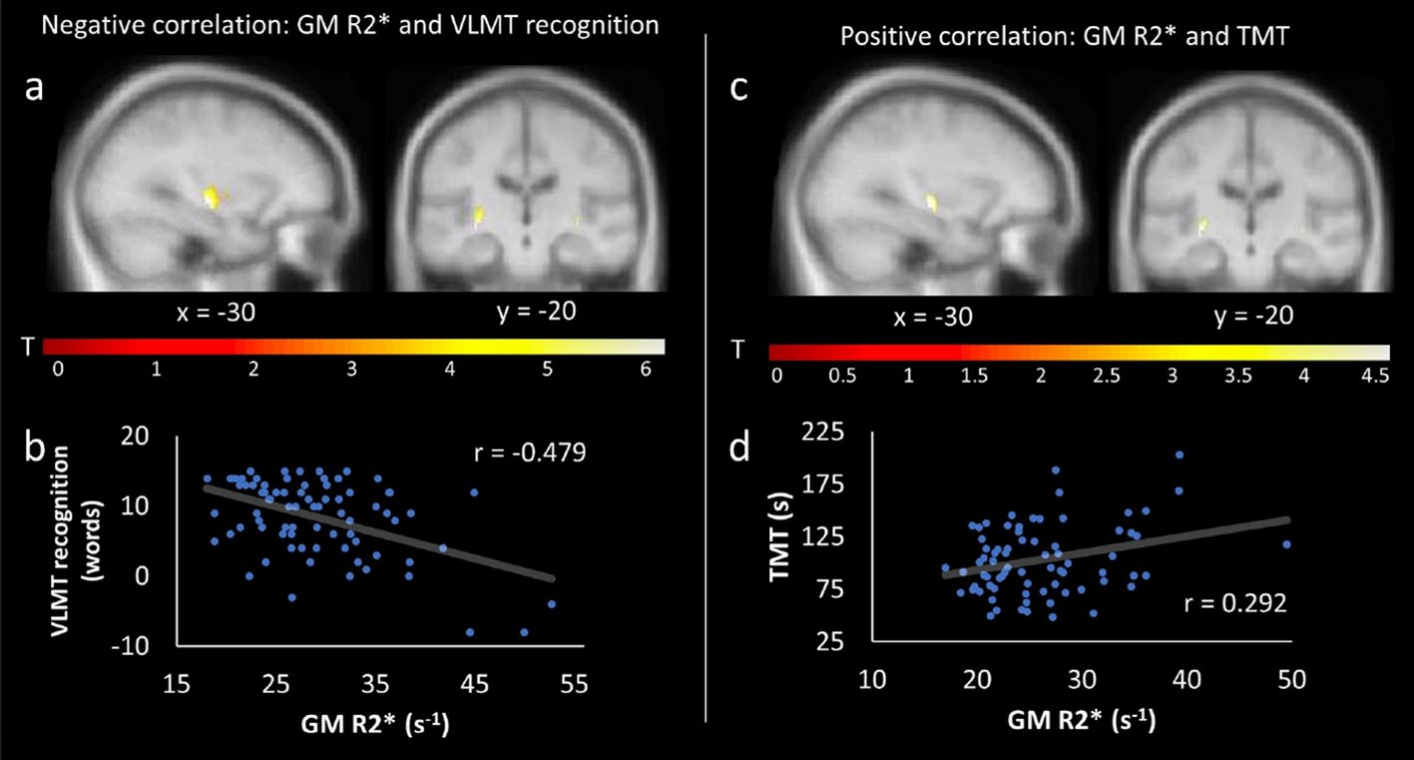
Relationship between cognitive performance and GM R2*. SPMs showing a significant negative relationship between GM R2* and VLMT recognition within the left and right putamen (**a**); a positive relationship between GM R2* and TMT within the left and right putamen (**c**). The corresponding correlation plots are shown in (**b**,**d**). In (**d**) three outliers were removed from the post hoc analysis (R2* values > 3 SD). For display purposes, SPMs were thresholded at p < 0.001, uncorrected, and superimposed on the mean T1-weighted image.

Regarding behavioral performance, GM R2* correlated negatively with performance in VLMT recognition (i.e., left and right putamen) and free recall (i.e., left and right putamen/pallidum) – cluster level FWE-corrected, p < 0.05 (Table 1; Fig. 4a-b). Furthermore, GM R2* of the left and right putamen correlated positively with the TMT (higher values [s] in the TMT imply worse performance; Table 1; Fig. 4c-d). There were no other significant effects with regard to R2*. When adding age as a covariate in a post hoc partial correlation, the effects remained significant (GM R2* and VLMT recognition: r = -0.400, p < 0.001; GM R2* and TMT: r = 0.240, p = 0.041).

To further break down different components of the TMT, regression analyses for TMT-A, TMT-B, TMT difference, and TMT ratio were performed. As a result, three clusters for TMT-B and one cluster for TMT difference (positive correlation) were revealed (Table 1). No significant effects were found for TMT-A and TMT ratio.

No significant correlates between structure and cognition could be observed on subsequent whole brain analysis (FWE, p < 0.05, k = 50 voxel).

### VBQ analysis on MT

Similar to the analyses above, a multiple regression analysis on GM MT was performed, including the covariates age, LPS 50 + , MWT, VLMT learning, VLMT cons, VLMT free recall, digit span, and d2-R. Due to outliers/missing values in the VLMT recognition and TMT, two additional regression analyses were conducted for age and VLMT recognition, as well as age and TMT. Therefore, three separate regression analyses were performed; the effects were small volume corrected, using a BG mask (cluster level FWE, p < 0.05; see “Materials and methods”).

**Figure 5:**
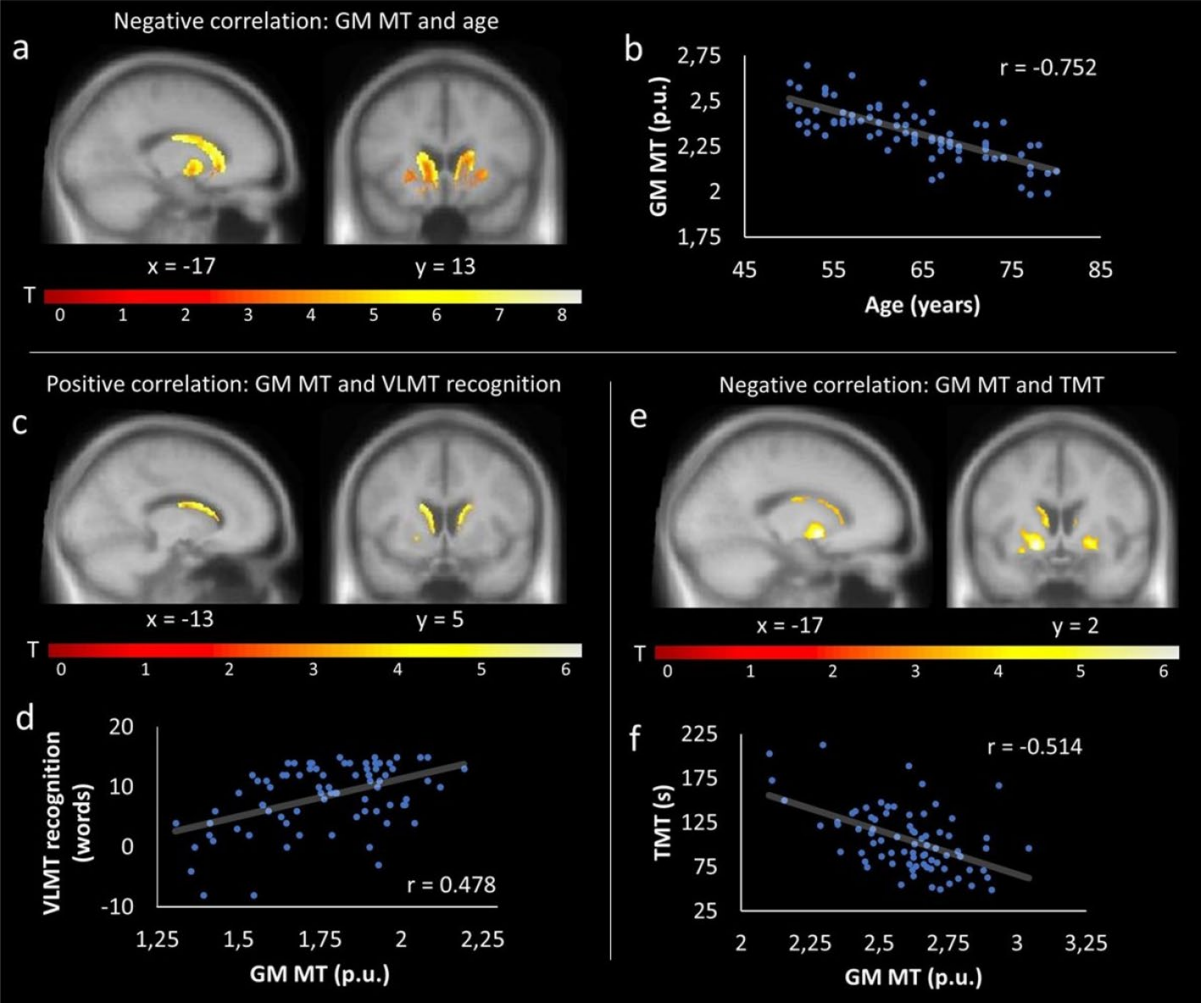
Relationship between GM MT, age, and cognitive performance. SPMs showing a significant negative relationship between GM MT and age within left and right caudate (**a**); a positive relationship between GM MT and VLMT recognition within the left and right caudate (**c**); and a negative relationship between GM MT and TMT within the left and right pallidum (**e**). The corresponding correlation plots for the clusters are shown in (**b**,**d**,**f**). For display purposes, SPMs were thresholded at p < 0.001, uncorrected, and superimposed on the mean T1-weighted image.

The analyses revealed negative correlations between age and MT within the BG (SVC). Three clusters within the caudate and pallidum were identified (Table 1; Fig. 5a-b). Regarding cognitive performance, GM MT correlated positively with VLMT recognition (Table 1; Fig. 5c-d) and GM MT in the left pallidum correlated positively with VLMT free recall (Table 1). Additionally, GM MT within the caudate, putamen, and pallidum correlated negatively with the TMT (higher values [s] in the TMT imply worse performance; Table 1; Fig. 5e-f). In a post hoc partial correlation with age as a covariate, all correlations remained significant (GM MT and VLMT recognition: r = 0.276, p = 0.015; GM MT and TMT: r = −0.274, p = 0.017).

Post-hoc regression analyses for the TMT revealed three clusters for TMT-A, seven clusters for TMT-B, and one cluster (negative correlation) for TMT differences (Table 1). There was no effect for TMT ratio. Finally, no significant correlates between structure and cognition could be observed on subsequent whole brain analysis (FWE, p < 0.05, k = 50 voxel).

### Analysis of the SN/VTA in native space

**Figure 6:**
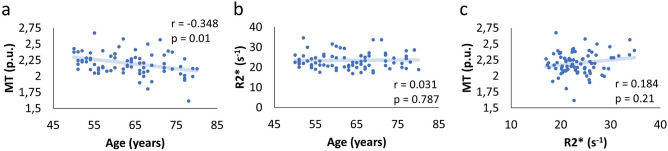
Relationship between MT, R2*, and age within the SN/VTA. We observed a significant negative correlation between MT and age (**a**); but no significant correlations between (**b**) R2* and age or (**c**) MT and R2* (p > 0.05). Benjamini-Hochberg (FDR) p-value adjustment was applied.

In a last step, individually defined ROIs for the SN/VTA in native space were used to extract MT and R2* values for each subject (mean MT [p.u.] = 2.2 ± 0.174; mean R2* [s^−1^] = 23.4 ± 4.01). A correlation analysis between age, R2*, MT, and each neuropsychological test revealed a negative relationship between age and MT within the SN/VTA (r = −0.348, FDR adjusted p = 0.01; Fig. 6a), but not between age and R2* (r = 0.031, FDR adjusted p = 0.787; Fig. 6b) or MT and R2* (r = 0.184, FDR adjusted p = 0.21; Fig. 6c). Furthermore, a positive correlation between SN/VTA R2* and TMT reached trend level (FDR adjusted p = 0.06; Table 3) – note that higher values (s) in the TMT imply worse performance.

Finally, a comparison between the correlations within the BG vs. SN/VTA was computed (see methods). All comparisons reached significance (p < 0.05), except from the TMT in the R2* analysis (p = 0.838; Table 2).

**Table 2:** Comparison between correlations within BG vs. SN/VTA. Significant results are highlighted with an asterisk.

		Age	VLMT recognition	TMT-sum
R2*	Z	3.466	−2.808	0.205
	p-value	< 0.001*	0.005*	0.838
MT	Z	−4.599	2.578	−3.587
	p-value	< 0.001*	0.010*	< 0.001*

### Relationship between age and cognitive performance

As expected, age correlated negatively with LPS 50 + , VLMT learning, VLMT free recall, and VLMT recognition, d2-R, and TMT (Table 3). Instead of TMT-sum, TMT-diff was included in the analysis, since it offers increased sensitivity to executive functioning; moreover, post-hoc MRI regression analysis with TMT-diff (but not TMT-ratio) became significant (Table 1).

**Table 3:** Correlation matrix for SN/VTA parameters. Age, R2*, and MT were correlated with each neuropsychological test (Pearson correlation). Benjamini-Hochberg (FDR) p-value adjustment was applied. Significant results are highlighted with an asterisk. Note that there was one missing value for VLMT recognition and that two participants had to be removed from the TMT-diff analysis (> 3 SD, see text).

		Age	R2*	MT
Age	r	–		
	p-value	–		
R2*	r	0.031	–	
	p-value	0.787	–	
MT	r	−0.348	0.184	–
	p-value	0.008*	0.211	–
MWT	r	0.123	−0.064	−0.137
	p-value	0.382	0.637	0.361
LPS 50+	r	−0.328	−0.093	0.126
	p-value	0.014*	0.499	0.382
VLMT learning	r	−0.432	−0.120	0.159
	p-value	0.001*	0.382	0.285
VLMT cons	r	0.228	0.048	−0.094
	p-value	0.140	0.697	0.499
VLMT recall	r	−0.418	−0.172	0.194
	p-value	0.001*	0.244	0.198
VLMT recognition	r	−0.416	−0.225	0.187
	p-value	0.001*	0.140	0.211
Digit span	r	−0.220	−0.146	0.081
	p-value	0.140	0.330	0.550
d2-R	r	−0.456	−0.124	0.203
	p-value	< 0.001*	0.382	0.183
TMT-diff	r	0.378	0.225	0.052
	p-value	0.004*	0.140	0.697

### Mediation analysis

**Figure 7:**
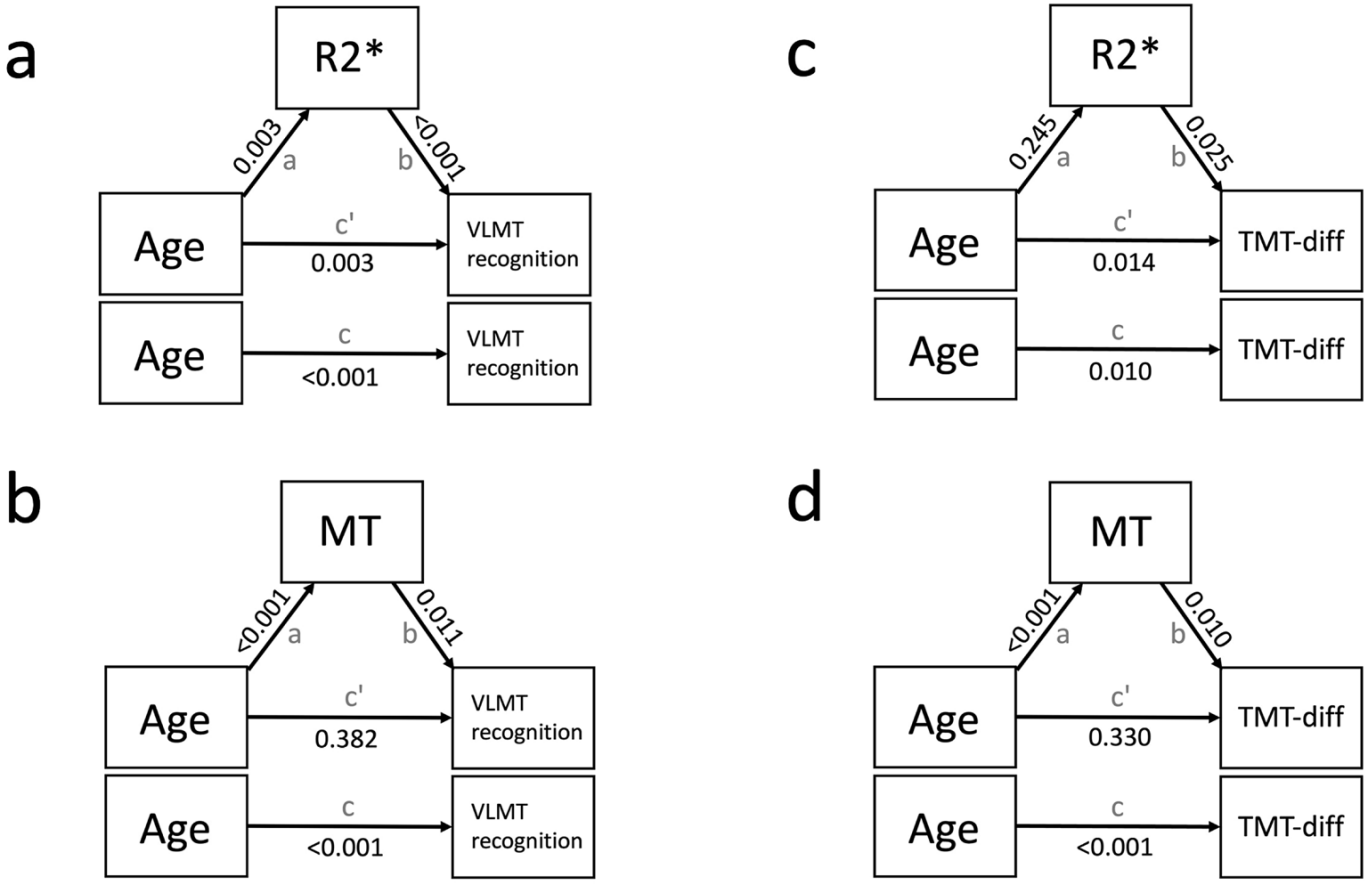
Illustration of the mediation model and its path estimates. Age served as predictor, VLMT recognition (**a,b**) and TMT-diff (**c,d**) as dependent variables, and R2* and MT analysis as mediator. Arrows indicate the relationship between the direct pathway (**c**'), and the indirect pathways (**a,b**). The total pathway (**c**) reflects the correlation between the predictor and the dependent variable. P-values are displayed on the arrows.

In a next step, a mediation analysis for significant structure-behavior correlates within the R2* and MT analysis (Fig. 7, Table 4) was performed, to further investigate the interplay between brain structure, cognitive performance, and age. In our models, age served as predictor, cognition (VLMT recognition and TMT-diff, respectively) as dependent variable, and brain structure (R2* and MT, respectively) as mediator. The analysis revealed that brain structure partially mediated the effects of age on cognition for both, R2* and MT. For the analysis of R2* and cognition, the indirect path mediated the effects of iron on verbal memory (Fig. 7a) to a degree of 29.80%, but failed significance for executive functioning (Fig. 7c; Table 4). The direct pathway via age explained 70.21% and 89.12%, respectively (Table 4). For the MT analysis, the indirect path mediated the effects of MT on verbal memory (Fig. 7b) to a degree of 68.50% and on executive functioning (Fig. 7d) to a degree of 64.24% (Table 4). In contrast, the direct pathways via age failed significance (Table 4). The R2* and MT analyses with TMT-sum score revealed congruent results (see supplements).

**Table 4:** Mediation estimates. Age served as predictor, VLMT recognition and TMT-diff as dependent variables, and R2* and MT as mediator. The labels a and b represent the indirect pathway, while c' represent the direct pathway.

Effect	Label	Estimate	SE	Z	p-value	% Mediation
R2* and VLMT recognition						
Indirect	a x b	−0.079	0.033	−2.370	0.018	29.80
Direct	c'	−0.186	0.063	−2.929	0.003	70.21
Total	c' + a x b	−0.264	0.065	−4.038	< 0.001	100
**MT and VLMT recognition**						
Indirect	a x b	−0.181	0.074	−2.456	0.014	68.50
Direct	c'	−0.083	0.095	−0.875	0.382	31.51
Total	c' + a x b	−0.264	0.065	−4.038	< 0.001	100
R2* and TMT-diff						
Indirect	a x b	0.085	0.082	1.033	0.302	10.88
Direct	c'	0.697	0.284	2.458	0.014	89.12
Total	c' + a x b	0.782	0.290	2.693	0.007	100
**MT and TMT-diff**						
Indirect	a x b	0.650	0.265	2.449	0.014	64.24
Direct	c'	0.361	0.371	0.975	0.330	35.76
Total	c' + a x b	1.011	0.282	3.581	< 0.001	100

## Discussion

We investigated age-related differences in GM and microstructural markers of myelin (i.e., MT) and iron (i.e., R2*) levels within the BG and their link to cognitive performance. In line with our hypotheses, cognitive performance negatively correlated with age in most tests. At the neural level, we observed age-related declines of GM volume and MT, and an increase of R2* relative to the tested age range. Importantly, performance in verbal memory and executive function was predicted by MT (positive relationship) and R2* (negative relationship). Finally, a ROI-analysis of the SN/VTA revealed age-related demyelination (as indicated by MT) but no clear link between behavior and R2* or MT, respectively. As such, our findings are compatible with the role of the BG in multiple cognitive functions, and they give new insights into the specific functional consequences of age-related microstructural changes.

Neural degeneration within the BG is typical for heathy aging^27,30,85^, and might reflect a loss of neuronal and dendritic architecture, rather than a loss of neurons^86,87^. Although the underlying processes are still unclear, microstructural changes and oxidative damage might be of particular relevance (see as a review^86^). Indeed, apart from GM reductions, our findings show that MT negatively correlated with age within the caudate and pallidum (Fig. 5a-b). This presumably indicates less macromolecular content (e.g., oligodendrocytes) and might reflect demyelinating processes^26,88^. A positive correlation between age and R2* within the putamen were accompanied by a negative relationship to MT (Fig. 3). This is in accordance with previous studies^3^, and may indicate a dysfunction of myelin-forming oligodendrocytes, which are sensitive to oxidative stress^26^, possibly triggered by increased iron levels ^13,24^. Importantly, the negative relationship between R2* and MT did not remain significant when controlling for age in a partial correlation, suggesting that the interplay between iron and demyelination is not independent from age. Therefore, future studies should further investigate the relationship between all three factors ideally in a longitudinal design.

With regard to behavior, we can confirm our hypothesis^2,3^ by demonstrating a negative relationship between R2* and verbal memory performance within the putamen/pallidum (Table 1; Fig. 4a-b). In order to encode novel information into long-term memory, the hippocampal VTA-loop model suggests that a hippocampal novelty signal is sent to the DA neurons within the SN/VTA via a polysynaptic path including the subiculum, nucleus accumbens, and ventral pallidum before DA-neurons back-project to the hippocampus^89,90^. While iron is required for DA synthesis^14^, an accumulation of it can impair DA production^8,14^. Therefore, increased iron levels within the bilateral putamen/pallidum may account for an imbalance of the loop, leading to impairments in verbal memory.

Importantly, while previous work has already demonstrated a relationship between striatal iron and episodic memory^2,3,91^, our findings show that iron accumulation (as indicated by R2*) within the BG also impact on executive functioning (as tested by the TMT) in healthy older adults (Fig. 4c-d). Psychological models of executive functioning typically include several mental processes, such as planning, task switching, inhibition, and cognitive flexibility (see e.g.,^92^). At the neural level, executive processing is strongly related to the medial frontal lobe^93^, but there is also a body of evidence for a close link to the fronto-striatal circuit^94–98^. Notably, in Parkinson’s disease, which is characterized by increased iron levels within the SN/VTA, red nucleus, putamen, pallidum, and caudate (e.g.,^15^), executive dysfunction is already present in early stages of the disease^99,100^. It has been suggested that executive disorders in Parkinson’s disease originate from DA depletion within the striatum, resulting in a dysfunction of the fronto-striatal circuit^98,101,102^. More evidence comes from a PET study testing patients with mild Parkinson’s disease and healthy controls on a typical planning task (Tower of London). While both groups showed overlapping task-related activation patterns within the PFC, only healthy controls showed activation within the right caudate. In contrast, in patients with Parkinson’s disease, activation within the right hippocampus was increased, suggesting a compensatory shift towards the declarative memory network^103^. Therefore, increases in BG iron levels during healthy aging may impair fronto-striatal circuits, leading to reduced executive functioning.

Verbal memory and executive functioning could also be predicted on the basis of BG myelination (as indicated by MT; Fig. 5c-f). At the physiological level, age-related reduction of myelin may impair saltatory conduction at the nodes of Ranvier and thereby, decreased velocity of action potentials along the myelin sheaths^104,105^. This, in turn, could impair behavior – in our case verbal memory and executive functioning. Indeed, previous studies could demonstrate that myelin facilitates learning processes (see as a review^106^), while demyelination is related to a deceleration of processing speed in healthy aging^107^. Moreover, in mild cognitive impairment, demyelination is found within several brain regions^108,109^.

In terms of the different components of the TMT, both subtests (TMT-A and TMT-B), the TMT-diff score and TMT-ratio were investigated (see methods). For the R2* analysis, positive associations were revealed for the TMT-B subtest and TMT-diff score, suggesting a stronger functional link to executive/cognitive control (TMT-B) compared to the working speed component (TMT-A). In other words, higher values of R2* resulted in worse performance in executive functioning, corrected for working speed. For the MT analysis, negative associations were observed for both TMT subtests (TMT-A and TMT-B), as well as for the TMT-diff score. Again, this suggests that impairments of BG integrity impact on executive functioning rather than on working speed.

In the present study, we observed a negative correlation between age and GM volume within the right caudate, pallidum, and putamen (Fig. 2a-b), and a (weak) link between GM volume and verbal memory (i.e., VLMT recognition) in particular within the caudate nucleus. This is in line with previous work on age-related structural degeneration of the BG^30,85,110^, and the notion of a close relationship between caudate volume and associative memory^30^, intelligence^111^, cognitive flexibility^112^, and learning^113^. However, it should be noted that the relationship between GM volume and VLMT recognition was only borderline significant (p = 0.07) when adding age as covariate in a partial correlation. Again, this suggests that the interplay between BG integrity and cognitive performance (including verbal memory) is not independent from age. Indeed, in our mediation analyses (Fig. 7) the indirect effects of brain structure (MT and R2*) on cognition (VLMT recognition and TMT-diff) were statistically significant and explained additional variance on top of the direct effects via age. However, this was only true for age/R2*/VLMT, age/MT/VLMT and age/MT/TMT but not age/R2*/TMT where the indirect pathway was not statistically significant. While this could be due to a non-significant relationship specifically between age and R2* (Fig. 7c), it also indicates that different markers of structural brain integrity (MT vs R2*) allow different conclusions regarding the interplay of age, brain structure, and cognition. This is further supported by the fact that for the MT but not R2* analyses, only the indirect paths reached significance, suggesting a subordinated direct effect of age. Together, structural integrity of the BG mediates the direct effect of age on cognitive performance. However, the strength of this relationship, in particular the direct effect of age on cognitive performance, appears to depend on the imaging modality (R2* vs MT).

A ROI-analysis of the SN/VTA revealed a significant negative correlation between age and MT, but no significant effect between age and R2* (Fig. 6). These observations are compatible with previous studies in healthy older adults, which also indicate age-related MT decreases^22,78^ but no significant changes in iron^19^. With regard to the pattern observed in the BG, this suggests that, during healthy aging, myelin decreases are typical in both regions (BG and SN/VTA), whereas iron increases are only typical within the BG but not SN/VTA. Interestingly, in patients with Parkinson’s disease, iron increases have been reported in the BG and SN/VTA^15^. This suggests that BG iron increases as well as demyelination in the BG and SN/VTA are typical for healthy aging, whereas SN/VTA iron levels are specific to pathologic neurodegeneration^14,17^. However, since only older adults have been tested here (50–80 years), we cannot exclude the possibility that a group of young controls might have shown significantly lower iron levels in the SN/VTA. Evidence for a continuous increase of SN/VTA R2* over the life-span comes from a study testing people aged 18–85 years, showing a positive relationship between age and SN/VTA R2*^28^. Thus, it is likely, that a younger control group would have shown significant lower R2* values compared to our older cohort.

Despite a significant relationship between age and demyelination (as indicated by MT) of the SN/VTA, there was no clear link between structural integrity (MT or R2*) and behavior (Table 3). The only hint was based on a negative correlation between R2* and executive function (TMT-diff, uncorrected p = 0.048), but this did not survive Benjamini–Hochberg correction (adjusted p = 0.140). This absence is in contrast to previous work on SN/VTA integrity in healthy older adults demonstrating a link between MTR and verbal memory^22^, MT and reward-related reaction times^114^ as well as MT and learning in a go-no-go-task^78^. These apparently divergent findings may relate to differences in MR data-acquisition protocols or differences in cognitive/behavioral readouts and they may suggest that the relationship between SN/VTA integrity and behavior may be modulated by other factors that were not explicitly included in our analysis (e.g., personality traits).

A direct comparison of the correlations between structural integrity and cognitive performance in the BG vs. SN/VTA revealed statistically significant differences (except from the correlation of TMT with R2*, Table 2). This means that the observed associations between age and R2*, age and MT, VLMT and R2*, VLMT and MT, as well as TMT and MT were significantly more pronounced within the BG as compared to the SN/VTA. Taken together with the absence of any significant effects outside of the BG in the whole brain analyses, this finding further underscores that the associations between structural integrity (R2* and MT) and cognitive functions (verbal memory and executive functioning) within the BG were region specific effects.

Finally, our findings must be interpreted with several limitations. First, MT and R2* are only indirect makers of myelin and iron contents and therefore, should be interpreted with caution (e.g.,^24^). However, post mortem studies provide histological evidence for a strong relationship between MT and myelin^21^ as well as R2* and iron^38^. Along the same lines, R2* is sensitive to both, iron and myelin^115,116^ but previous work suggests that the correlation between R2* and iron concentration in GM might be higher as compared to R2* and iron concentration in WM^38^. In any case, the interpretation of R2* is not only indirect but also complex (see e.g.,^74,117^). Second, VBM might be vulnerable to include voxels of WM, CSF, or blood vessels and can, therefore, confound estimates of R2* and MT (see^118^). Manual segmentation of the SN/VTA, on the other hand, might have been biased by iron concentration^70^. Third, we would like to point out that the current study followed a cross-sectional but no longitudinal approach. Therefore, terms like “increase” or “decrease” relate to between-subjects comparisons within the age range of our sample but they do not imply individual development over time. Moreover, separate regression models for VLMT recognition and TMT were performed due to missing data. Therefore, one could argue that the observed effects of both models are due to reduced complexity. However, for verbal memory performance, correlations were also observed for VLMT recall in the multiple regression models, which speaks for a robust finding. Finally, future studies should more thoroughly take underlying mechanisms of age-related structural changes into account. In this view, hypertension and vascular diseases may relate to the brain’s microstructure and cognitive functioning. Indeed, hypertension is one of the main risk factors for stroke^119^ and small vessel disease^120^, both strongly linked to dementia^121–123^. Moreover, education^124^ and life-style factors^125^ have been shown to account for inter-individual differences in healthy aging.

To summarize, age-related markers of iron levels and demyelination within the BG were correlated, which is in line with the role of iron in dysfunctional myelin synthesis. Importantly, iron levels and demyelination predicted both verbal long-term memory and executive functioning, which gives novel insights into the behavioral consequences of BG microstructural changes. From a more general perspective, our results further suggest that increased iron and demyelination within the BG are typical for healthy aging and they might be distinguished from age-related differences in SN/VTA microstructure.

## Supplementary Information

Supplementary Information.

## Data Availability

The data that support the findings of this study are available on reasonable request from the corresponding authors (DB or NB). The data are not publicly available due to data security regulations by the local ethics committee.
