# Supplementary material for: Age-related iron accumulation and demyelination in the basal ganglia are closely related to verbal memory and executive functioning
Source: Sci Rep. 2021 May 3;11:9438. doi: 10.1038/s41598-021-88840-1 (PMC8093241; doi:10.1038/s41598-021-88840-1)
Supplement: Supplementary file 1 — Supplementary Information. [file 41598_2021_88840_MOESM1_ESM.docx]

**Title: Age-related iron accumulation and demyelination in the basal ganglia are closely related to verbal memory and executive functioning**

**Author names and affiliations**

Davina Biel^1, 2^, Tineke K. Steiger^1^, Nico Bunzeck^1, 3^

^1^ Department of Psychology, University of Lübeck, 23562 Lübeck, Germany

^2^ Institute for Stroke and Dementia Research (ISD), University Hospital, LMU Munich, 81377 Munich, Germany

^3^ Center of Brain, Behavior and Metabolism (CBBM), University of Lübeck, Ratzeburger Allee 160, 23562 Lübeck, Germany

**Supplements**

**TMT-sum**

**
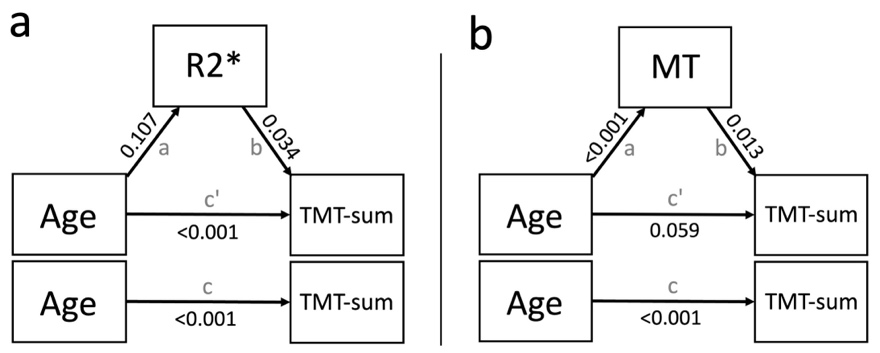
**

**Figure 1**. Illustration of the mediation model and its path estimates. Age served as predictor, TMT-sum as dependent variables, and R2* (**a**) and MT (**b**) analysis as mediator. Arrows indicate the relationship between the direct pathway (c'), and the indirect pathways (a+b). The total pathway (c) reflects the correlation between the predictor and the dependent variable. P-values are displayed on the arrows.

**Table 1.** Mediation estimates. Age served as predictor, TMT-sum as dependent variables, and R2* and MT as mediator. The labels a and b represent the indirect pathway, while c' represent the direct pathway.

| Effect | Label | Estimate | SE | Z | p-value | % Mediation |
| --- | --- | --- | --- | --- | --- | --- |
| R2* and TMT-sum | | | | | | |
| Indirect | a x b | 0.168 | 0.131 | 1.284 | 0.199 | 9.62 |
| Direct | c' | 1.578 | 0.429 | 3.679 | <0.001 | 90.38 |
| Total | c' + a x b | 1.745 | 0.434 | 4.021 | <0.001 | 100 |
| MT and TMT-sum | | | | | | |
| Indirect | a x b | 1.004 | 0.419 | 2.399 | 0.016 | 48.16 |
| Direct | c' | 1.081 | 0.572 | 1.888 | 0.059 | 51.84 |
| Total | c' + a x b | 2.085 | 0.423 | 4.925 | <0.001 | 100 |
